# Minimally-invasive lateral thoracic and lumbar interbody fusion (LLIF) with expandable interbody cages – Considerations, complications & outcomes

**DOI:** 10.1016/j.bas.2024.102870

**Published:** 2024-07-15

**Authors:** Martin N. Stienen, Gregor Fischer, Linda Bättig, Anand Veeravagu, Benjamin Martens

**Affiliations:** a Spine Center of Eastern Switzerland, Cantonal Hospital of St. Gallen & Medical School of St. Gallen, St. Gallen, Switzerland; b Department of Neurosurgery, Cantonal Hospital of St. Gallen & Medical School of St. Gallen, St. Gallen, Switzerland; c Department of Neurosurgery, Stanford University, Stanford, CA, USA; d Department of Orthopedic Surgery, Cantonal Hospital of St. Gallen & Medical School of St. Gallen, St. Gallen, Switzerland

**Keywords:** Lateral lumbar interbody fusion, Lateral thoracic interbody fusion, Expandable spacer, Complications, Sagittal parameters, Outcome

## Abstract

**Introduction:**

Reports about lateral lumbar or thoracic interbody fusion (LLIF) using expandable interbody spacers are sparse.

**Research question:**

To report our experience with the use of expandable spacers for LLIF.

**Material and methods:**

We reviewed all consecutive LLIF patients with use of an expandable titanium interbody implant (ELSA® Expandable Integrated LLIF Spacer, Globus Medical Inc, PA (USA)) between September 2018 and January 2024.

**Results:**

We identified 503 patients, in which we performed LLIF at 732 levels. In 63 patients (12.5%) and 70 levels (9.6%) an expandable spacer was used. The mean age was 61.4 years, 57.1% were females. LLIF was performed between T11/12 – L4/5 in the setting of fusion procedures (mono-/bisegmental (20; 28.6%), 3–7 segments (29; 41.4%); >7 segments (21; 30.0%)), of which 21 (33.3%), 20 (31.8%) and 22 (34.9%) were for traumatic, deformity/revision and other diagnoses. Surgery included release of the anterior longitudinal ligament in 30 cases (42.9%). Intraoperative adverse events (AEs) were noted in 2 (3.2%), postoperative AEs in 27 (42.9%) at discharge, 17 (27.0%) at 3 months and 14 (22.2%) at 12 months. Segmental sagittal Cobb angle changed from 1.3° (preoperative) to 13.0° at discharge (p < 0.001), 12.7° at 3 months (p < 0.001) and 13.3° at 12 months (p < 0.001). Functional outcome was excellent/good in 43 (68.3%; 5 missing) at 3 months and in 37 (58.7%; 10 missing) at 12 months.

**Discussion and conclusion:**

The use of LLIF with an expandable spacer was safe, promoted solid fusion and enabled powerful correction of sagittal segmental Cobb angle, which was maintained during follow-up.

## Highlights

• LLIF is increasingly considered for anterior column reconstruction.
• We reviewed consecutive patients treated by LLIF and use of an expandable spacer.
• We identified 63 LLIF patients, mostly with revision surgery, deformity, or trauma.
• Segmental Cobb angle changed from 1.3° (preop) to 13.3° at 12 months (p < 0.001).
• LLIF with an expandable spacer was safe & enabled correction of sagittal deformity.

## 1 Introduction

Lateral lumbar interbody fusion (LLIF) using a minimally-invasive, retroperitoneal, trans- or anterior to psoas (ATP) approach to employ an interbody cage is increasingly utilized for segmental lordosis correction, indirect decompression and fusion promotion. It offers the advantages of preserving segmental stabilizing elements, e.g., the anterior and posterior longitudinal ligaments, ligamentum flavum, and the facet joints while reducing the risk of direct nerve root injury as experienced in posterior interbody fusion. LLIF allows for accurate endplate preparation and insertion of a spacer with large footprint and the desired degree of lordosis (Huo et al., 2023; Smith et al., 2010). If required, LLIF can be applied to the thoracolumbar junction via lateral opening of the diaphragm, or to the lower thoracic levels via thoracotomy.

Recently, expandable spacers for LLIF have been introduced, but reports about their use remain scarce and often only entail case reports or smaller series (Hiyama et al., 2023; Huo et al., 2023; Li et al., 2020; Macki et al., 2021; Omosor et al., 2023). Expandable LLIF spacers are designed to offer the advantage of controlled *in-situ* expansion, which can help to optimize spacer-positioning and endplate protection and may lead to increased fusion rates, reduced subsidence rates and improved outcome compared to static cages (Huo et al., 2023; Li et al., 2020). As adjustable hyperlordotic spacers up to 30° are available, LLIF in conjunction with release of the anterior longitudinal ligament (ALL) offers a powerful method for anterior column realignment (ACR) and segmental correction of spinal deformities. In the setting of trauma, hyperlordotic LLIF cages may be used to compensate for kyphosing vertebral body fractures (AO Spine types A1, A3 & A4), or to close wide anterior gaps in extension fractures (AO Spine type B3) while promoting fusion. Again, literature on the use of LLIF in trauma patients is extremely limited and much more frequently deals with corpectomies than with interbody fusion as a treatment option to achieve monosegmental fusion (Amaral et al., 2013; Meredith et al., 2013; Podet et al., 2020; Roblesgil-Medrano et al., 2022; Smith et al., 2010; Walker et al., 2018).

We set out to review our series of patients treated with LLIF and use of an expandable cage with the intention to critically analyze the patient conditions, various surgical parameters, intra- and postoperative adverse events (AEs), radiological and clinical outcomes.

## 2 Material and methods

### 2.1 Hospital setting

In our academic, tertiary teaching-hospital about 1100–1300 spine surgical procedures under general anesthesia are performed annually. LLIF with static cages was introduced in November 2011 and is performed about 50–60 times annually. The ELSA® Expandable Integrated LLIF Spacer (Globus Medical Inc, PA, USA) as expandable option for LLIF was introduced in September 2018. LLIF procedures were performed by nine senior spine surgeons, or under supervision by fellows or senior residents.

### 2.2 Patient identification

Unique patient numbers (UPNs) of patients, where an ELSA® spacer was employed until January 2024 were identified by electronic review of our hospital's purchasing department. In addition, we cross-checked the operating program for any LLIF procedures to make sure those with an expandable implant were included. Types of procedures included ventro-dorsal, dorso-ventral and dorso-ventro-dorsal fusion procedures. No stand-alone procedures were performed.

### 2.3 Data collection and variables

Three surgeons (LB, GF, MNS) retrospectively reviewed the electronic patient charts to extract relevant information. Baseline information included age, sex, body measures, smoking status, ASA grade, Charlson Comorbidity Index, (Charlson et al., 1987) and the Canadian clinical frailty scale (ranging from 1 (very fit) to 9 (terminally ill)), (Rockwood et al., 2005) as well as the criteria for surgery (e.g., trauma, deformity, revision) and the AO Spine classification for thoracolumbar trauma for traumatic cases (Vaccaro et al., 2013).

Baseline sagittal segmental Cobb angle (segmental lordosis (SL); e.g., upper endplate of L3 vertebra to lower endplate of L4 vertebra for the L3-L4 segment), as well as spino-pelvic parameters including pelvic incidence (PI), lumbar lordosis (LL; from L1-S1), pelvic tilt (PT) and C7-sagittal vertebral axis (C7 SVA) were measured using standing scoliosis x-ray images, whenever available. The Roussouly type of spinal geometry and ideal LL were calculated using a web-based app (http://www.spinebit.io), which is based on the formulas by Le Huec and the European Spine Study Group (Laouissat et al., 2018; Pizones et al., 2020; Sebaaly et al., 2018).

Surgical parameters included the number of segments fused (e.g., T10-S2Ai), LLIF segment (e.g., L3-L4), intraoperative release of the ALL, length of surgery (in minutes), estimated blood loss (EBL; in ml; defined by anesthesiologists from suction drainage and bloody swabs), and intraoperative AEs.

Postoperative AEs at time of discharge from hospital were considered, as documented by the discharge report, classified according to the Therapy-Disability-Neurology (TDN) scoring system (Terrapon et al., 2021). The same was recorded at 3 months (3M; AEs occurring between discharge and 3M follow-up) and at 12 months (12M; AEs occurring between 3M and 12M follow-up). Clinical outcome was graded according to Macnab into four categories, as best estimated from the follow-up letter (excellent, good, fair, poor). (Joswig et al., 2016; Macnab, 1971; Tafazal and Sell, 2006).

Postoperative radiological outcomes at 3M and 12M again included the sagittal segmental Cobb angle, LL, PI, PT and C7 SVA. Pseudarthrosis at the LLIF level was defined as failed attempt of spinal fusion, manifesting with axial or radicular pain weeks to months after the index operation (Raizman et al., 2009). Diagnosis of pseudarthrosis was based on both the clinical presentation in conjunction with available imaging studies (x-ray, computed tomography (CT) and Single Photon Emission Computed Tomography (SPECT)), after ruling out other causes of persistent pain.

### 2.4 Indication & surgical technique

LLIF was considered for a wide range of indications, including degenerative disc disease with or without spondylolisthesis, revision surgery, deformity and trauma. Reasons to choose LLIF over posterior approaches included virgin access (e.g., in revision cases), diminished bone quality and need for a wide-footprint interbody spacer spanning the ring apophysis (e.g., in patients with osteoporosis), possibility for indirect decompression, degenerative scoliosis that can be corrected at large parts via LLIF interbody spacers, need for a hyperlordotic implant (e.g., in trauma patients with kyphosing burst fractures or to correct degenerative deformities) and reduced operative time (e.g., in patients requiring multi-level interbody fusion). Expandable LLIF spacers were chosen over static LLIF cages when the intervertebral distance was wider than the largest static implant available (12 mm in our department), when the ALL was intentionally released to avoid implant migration into the retroperitoneal space/thoracic cavity (as the expandable device can be screw-fixated), when >15° of SL needed to be restored (as the expandable spacers are available in up to 30° of lordosis), and in situations where careful *in-situ* expansion of the implant was considered safer than forcing in a static implant (e.g., patients with diminished bone quality or unstable fracture types).

After positioning the patient strictly lateral under fluoroscopic control, we typically approach the disc space from the left side. In the lower lumbar spine (L4-L5 and L3-L4) an anterior-to-psoas or a trans-psoas approach utilizing directional electromyography (EMG) is used. The junctional level Th12-L1 may require a phrenicotomy, for the thoracic segments either a retropleural access without pleural opening or a mini-thoracotomy is used. After fluoroscopic confirmation of the correct location (and serial dilatation in the lumbar spine), the table-mounted MaXcess® retractor (NuVasive, Inc., San Diego, CA (USA)) is brought in and fixed firmly. The disc preparation is done with a combination of curettes and rongeurs; the Cobb elevator is used to release the contralateral anulus. If needed, the ALL is cut with a knife or a chisel after applying tension with an expandable sizer, thus optimizing controlled ALL release while protecting the blood vessels anterior of the spine. After trialing with a sizer for the antero-posterior and coronal planes, the expandable implant is brought in and opened under fluoroscopic control until it gains bony contact. It is fixed to one or both vertebral bodies with an integrated screw to prevent it from migrating anteriorly, especially when hyperlordotic implants are used, and the ALL has been released. The spacer can then be finally expanded until the desired implant height and lordosis is achieved. The graft chamber of the implant can then be filled with bone graft using a funnel. We do not perform stand-alone LLIF; all patients received additional percutaneous or open posterior pedicle screw instrumentation.

### 2.5 Statistical analysis

We used Stata (StataCorp LLC, College Station, TX (USA)) v14.2 for Mac and employed mostly descriptive statistics, reporting results as mean (standard deviation) or count (percent). For the analysis of sagittal spinal parameters over time, outcomes were compared to the preoperative value using paired t-tests. In patients undergoing LLIF surgery for spinal deformity (n = 16), we calculated the difference between desired (ideal) LL and observed (actual) LL, as well as PI-LL mismatch at each time point of follow-up. Probability values < 0.05 were considered statistically significant.

### 2.6 Ethical considerations

The institutional review board (IRB) of Eastern Switzerland approved the study (BASEC ID, 2023-01343). Retrospective collection, analysis and publication of anonymized patient data was allowed with an institutional waiver for informed consent.

## 3 Results

### 3.1 Patient cohort

We identified 503 patients, in which we performed LLIF at 732 levels. In 63 patients (12.5%) and 70 levels (9.6%) an expandable spacer was used, which built the sample for this analysis.

The mean age of the cohort was 61.5 years (SD 15.7), 57.1% were female and 25.4% active smokers. The cohort had overall moderate surgical risk (44.5% ASA grade III or higher), comorbidities (31.8% Charlson Comorbidity Index Score 4 or higher) and frailty scores (23.8% mildly to very severely frail). The applications for LLIF were thoracolumbar spine trauma in 33.3%, deformity/revision in 31.8%, degenerative in 25.4% and other in 9.5% of cases (including, e.g., spondylodiscitis). More detailed baseline demographic information can be found in Table 1.

**Table 1:** Baseline demographic information. Results are presented as mean (standard deviation, range) or count (percent). *According to the AO Spine thoracolumbar fracture classification, 8 (12.7%) were A3, 4 (6.4%) were A4, 1 (1.6%) were B1, 8 (12.7%) were B2 and 1 (1.6%) were B3 injuries types. SS = sacral slope.

Age in years	61.4 (15.7, 17–87)
Sex	
Female	36 (57.1%)
Male	27 (42.9%)
ASA risk scale	
I	14 (22.2%)
II	21 (33.3%)
III	27 (42.9%)
IV	1 (1.6%)
Charlson comorbidity index	2.6 (2.5, 0–9)
0–1	27 (42.9%)
2–3	16 (25.4%)
4 or higher	20 (31.8%)
Canadian clinical frailty index	
Very fit or well	19 (30.2%)
Managing well or vulnerable	29 (46.0%)
Mildly or moderately frail	13 (20.6%)
Severely or very severely frail	2 (3.2%)
Smoking status	
Active smoker	16 (25.4%)
Former smoker	6 (9.5%)
Nonsmoker	41 (65.1%)
Indication for surgery	
Trauma*	21 (33.3%)
Revision/Deformity	20 (31.8%)
Degenerative	16 (25.4%)
Other	6 (9.5%)
Roussouly type of spinal geometry	
Type 1 (SS < 35°)	7 (11.1%)
Type 2 (SS < 35°)	15 (23.8%)
Type 3 (35° < SS < 45°)	20 (31.8%)
Type 4 (SS > 45°)	21 (33.3%)
**Total**	**63 (100%)**

Table 2 summarizes the surgical parameters. LLIF was performed in the thoracic spine (T11-L1) in 16 (22.8%) and in the lumbar spine (L1-L5) in 54 (77.2%) cases. Most were rather lengthy (mean surgery time 362min), multiple-level fusion procedures (3–7 segments 41.4%; 8 or more segments 30.0%), often entailing intentional intraoperative ALL release (42.9%) and employing lordotic or hyperlordotic implants (85.7%).

**Table 2:** Surgical parameters. Results are presented as mean (standard deviation, range) or count (percent). ALL = anterior longitudinal ligament; LLIF = lateral lumbar or thoracic interbody fusion. * Complications: asymptomatic cement leakage in 2 patients and cerebrospinal fluid leak in 1 patient.

LLIF segment	
T11-12	4 (5.7%)
T12-L1	12 (17.1%)
L1-2	6 (8.6%)
L2-3	14 (20.0%)
L3-4	23 (32.9%)
L4-5	11 (15.7%)
Number of fused segments	4.7 (3.5, 1–18)
Mono-/bisegmental	20 (28.6%)
3–7 segments	29 (41.4%)
8 or more segments	21 (30.0%)
ALL release	
Yes	30 (42.9%)
No	40 (57.1%)
Length of surgery, in minutes	362 (166, 88–725)
Estimated blood loss, in milliliters	757 (747, 20–4000)
Type of interbody cage	
Parallel (0° lordosis)	5 (7.1%)
Anatomical (6° lordosis)	5 (7.1%)
Lordotic (5–20° lordosis)	47 (67.1%)
Hyperlordotic (15–30° lordosis)	13 (18.6%)
Intraoperative AEs	
No	61 (96.8%)
Yes, type:	2 (3.2%)
Vascular injury	–
Nerve injury	–
Cage subsidence	–
Other*	2 (3.4%)
**Total**	**63 patients/70 levels (100%)**

### 3.2 Follow-up and reasons for missing data

Patients were discharged after a mean of 12.1 (SD 8.5) days. The 3M follow-up was completed by 58 (92.1%) patients at a mean of 93 (SD 30) days postoperative. The 12M follow-up was completed by 53 (84.1%) patients at a mean of 425 (SD 237) days postoperative. Reasons for drop-out during follow-up included mostly surgery performed within <1 year, patients moving away from the area (e.g., trauma patient living abroad) or seeking further care at another facility.

The mechanism behind missing data was missing at random. No imputation of missing data was performed as the missing data burden was low, and we did not apply “last-value-carried-forward” methods for their well-known flaws (Sterne et al., 2009).

### 3.3 Radiological outcomes

Table 3 illustrates the evolution of the sagittal radiological parameters over time. There was a significant postoperative increase in LL and SL, as well as a significant decrease in both PT and C7 SVA at discharge, which was preserved over time.

**Table 3:** Sagittal radiological parameters over time. Results are presented in degree (°) or centimeters (cm) as mean (standard deviation, range). The level of significance is indicated, comparing the value at follow-up with the preoperative value. ALL = anterior longitudinal ligament; C7 SVA = C7 sagittal vertical axis; LL = lumbar lordosis; PI = pelvic incidence; PT = pelvic tilt.

Parameter	Preoperative	Discharge	3 months	12 months
PI, in °	53.0 (12.6)	–	–	–
LL, in °	32.7 (18.5)	45.9 (9.2) p < 0.001	45.3 (13.4) p < 0.001	43.3 (15.0) p < 0.001
PT, in °	23.6 (10.3)	18.8 (7.7) p < 0.001	20.7 (9.7) p = 0.040	19.6 (9.1) p = 0.003
Segmental lordosis, in °	1.3 (16.0)	13.0 (13.1) p < 0.001	12.7 (13.8) p < 0.001	13.3 (14.5) p < 0.001
with ALL release	−2.8 (13.6)	16.4 (13.5), p < 0.001	15.0 (13.8), p < 0.001	16.9 (14.0), p < 0.001
without ALL release	4.3 (17.1)	10.5 (12.3), p = 0.006	10.9 (13.7), p = 0.029	10.4 (14.4), p = 0.131
C7 SVA, in cm	8.0 (6.4)	5.6 (4.3) p = 0.001	4.9 (4.2) p = 0.005	6.6 (5.1) p = 0.114
**Total**	**n** = **63 patients/n** = **70 levels (100%)**			

**Fig. 1:**
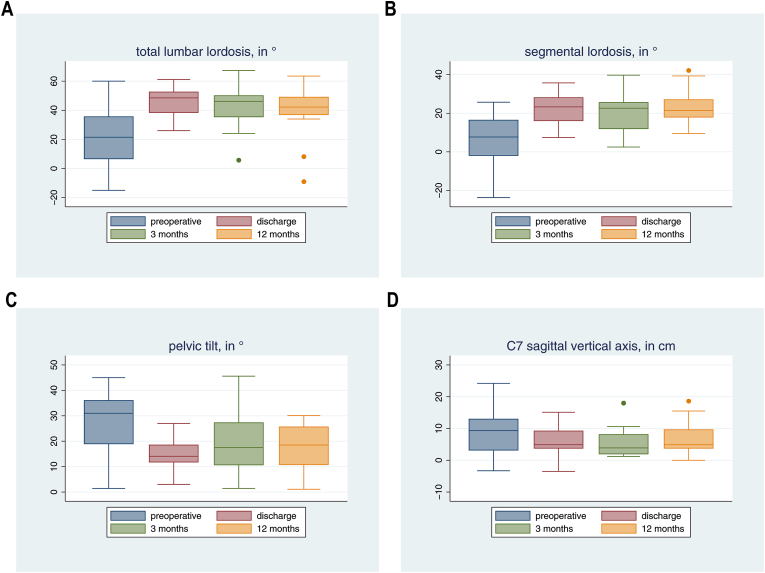
Total lumbar lordosis (LL; **A**) and segmental lordosis (SL; in °; **B**), as well as pelvic tilt (PT; in °; **C**) and C7 sagittal vertical axis (SVA; **D**) values (in cm) in the subgroup of n = 16 patients undergoing surgery for adult spinal deformity. LL increased from preoperative to discharge (average + 24.0°; p < 0.001) and remained increased at 3 months (average + 18.6; p = 0.006) and at 12 months (average + 14.0; p = 0.052). SL increased from preoperative to discharge (average + 17.0°; p < 0.001) and remained increased at 3 months (average + 14.4; p = 0.001) and at 12 months (average + 15.7; p = 0.002). PT decreased by 11.9° at time of discharge (p = 0.001) and remained decreased at 3 months (average −6.5°; p = 0.095) and at 12 months (average −9.1; p = 0.003). C7 SVA decreased by 4.1 cm at time of discharge (p = 0.043) and tended to remain decreased at 3 months (average −3.8 cm; p = 0.177) and at 12 months (average −2.7 cm; p = 0.250).

In patients with adult spinal deformity (n = 16), mean PI-LL mismatch was −29.6° (SD 23.1) before surgery, −5.6° (SD 12.0) at discharge, −8.4° (SD 17.8) at 3M and −9.9° (SD 19.3) at 12M. The difference between actual and ideal LL was −33.0° (SD 21.3) before surgery, −9.0° (SD 9.2) at discharge, −12.2° (15.5) at 3M and −12.9° (SD 15.5) at 12M. Lumbar and SL were significantly increased (all p < 0.05), while PT and C7 SVA values were decreased at discharge, 3M and 12M (Fig. 1A–D).

In patients where the ALL was released, the change in pre-to immediate postoperative SL was greater (−2.8 ± 13.6° vs. 16.4 ± 13.5°, p < 0.001) than in patients, where the ALL was not released (4.3 ± 17.1° vs. 10.5 ± 12.3°, p = 0.006).

### 3.4 Clinical outcomes

The rates of excellent or good outcome at 3M and 12M were 68.2% (5 patients missing) and 58.7% (10 patients missing), respectively (Table 4). Postoperative AEs were observed in 27 (42.9%) at discharge, 17 (27.0%) at 3M and 14 (22.2%) at 12M; detailed information on the AE type and severity is displayed in Table 4. None of the adverse events were device-related and no revision surgeries were required. We observed no pseudarthrosis at the LLIF level in any case during follow-up (Table 4).

**Table 4:** Clinical outcomes over time. Results are presented as count (percent). *Adverse events (AEs) are indicated as occurring between surgery and discharge, discharge and 3 months, or between the 3- and 12-month follow-up. **at the LLIF level. TDN = Therapy-Disability-Neurology grading scale of complications.

Parameter	Discharge	3 months	12 months
Functional outcome Excellent Good Fair Poor Missing data	–	22 (34.9%) 21 (33.3%) 9 (14.3%) 6 (9.5%) 5 (7.9%)	16 (25.4%) 21 (33.3%) 12 (19.1%) 4 (6.3%) 10 (15.9%)
Postoperative AE* No Yes Missing data	36 (57.1%) 27 (42.9%) - (0%)	41 (65.1%) 17 (27.0%) 5 (7.9%)	39 (61.9%) 14 (22.2%) 10 (15.9%)
TDN grading scale Grade 1 Grade 2 Grade 3 Grade 4 Grade 5 Missing data	- (0%) 15 (23.8%) 8 (12.7%) 4 (6.3%) - (0%) - (0%)	2 (3.2%) 5 (7.9%) 10 (15.8%) - (0%) - (0%) 5 (7.9%)	1 (1.6%) 2 (3.2%) 9 (14.3%) 1 (1.6%) 1 (1.6%) 10 (15.8%)
Pseudarthrosis** No Yes	–	63 (100%) - (0%)	63 (100%) - (0%)
**Total**	**n=63 (100%)**		

## 4 Discussion

In this series of 63 consecutive patients undergoing LLIF surgery with an expandable interbody spacer for various diagnoses, we found that by use of this technique we were able to effectively restore both SL and LL, decrease PT and C7 SVA values in the short- and long-term. The LLIF procedures were typically part of more extensive, often multi-level dorso-ventral, ventro-dorsal or dorso-ventro-dorsal fusion procedures for challenging underlying conditions. Despite this, the rates of intra- and postoperative AEs were reasonably low, and the majority of patients reported a good or excellent outcome at the 3- and 12-months follow-up. The fusion rate at the LLIF segments were 100% at the 12-months follow-up.

### 4.1 Considerations for LLIF surgery with an expandable spacer

The cohort was heterogenous, as we focused more on the operative technique than on strictly defined diagnostic criteria in this article. Hence, we analyzed patients undergoing this type of procedure for underlying deformity, spinal trauma, or other conditions including degenerative disc disease and pseudarthrosis, which allows for some in-depth analysis of potentially beneficial applications for LLIF.

**Fig. 2:**
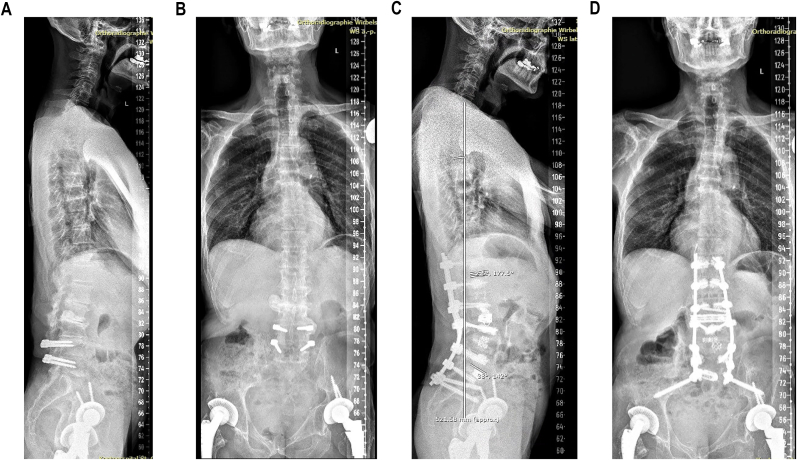
Typical case vignette of a 65-year-old male with history of prior dynamic stabilization at L4-L5 15 years ago, kyphotic autofusion of the L3-L4 segment and largely distorted spinal geometry. He was treated by lateral lumbar interbody fusion of L1-L3 with anterior column realignment (ACR) at L2-L3 with deliberate release of the ALL and lengthening of the anterior column by the 60 mm expandable lateral interbody cage (5–20° lordotic), combined with posterior osteotomies and compression. For the fused segments L3-L5 a pedicle subtraction osteotomy was applied. **A**: Sagittal scoliosis x-rays preoperative. **B**: Coronal scoliosis x-rays preoperative. **C**: Sagittal scoliosis x-rays 12 months postoperative. **D**: Coronal scoliosis x-rays 12 months postoperative.

In deformity patients, LLIF was considered for non-fused spinal segments where either the aim was to restore LL (for reason of more effective correction and restoration of lordosis at the correct level in terms of spinal geometry according to the Roussouly type) or where interbody spacers were needed at multiple levels (for reasons of reduced operative time). Especially the possibility of deliberate release of the ALL and lengthening of the anterior column combined with posterior osteotomies and compression offers the opportunity for well controlled ACR, which may help mitigate the need for shortening three-column osteotomies (Leveque et al., 2017; Mundis et al., 2017; Saigal et al., 2016; Xu et al., 2018). In our patients with ALL release, the mean change in SL was 19.2° (SD 9.4°) versus 6.1° (SD 13.5°) in patients where the ALL was not released. A typical case vignette is illustrated in Fig. 2A–D.

**Fig. 3:**
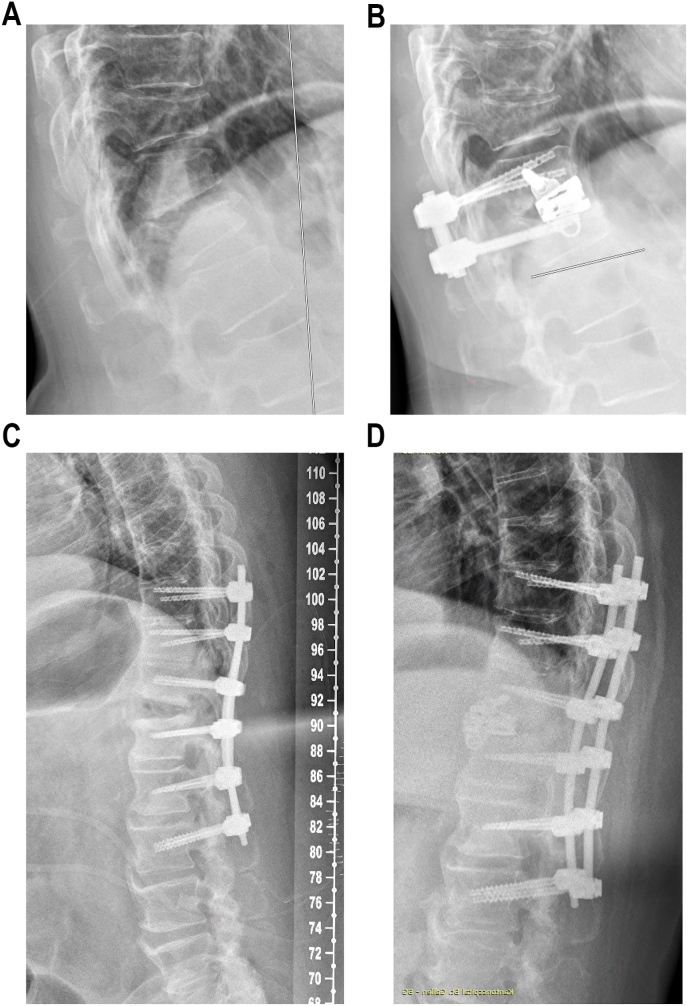
Typical case vignette of a 62-year-old female with history of prior, healed atraumatic T10 fracture (OF Type 2) and acute, kyphosing incomplete burst fracture (AO Spine Type A3) of Th12. **A**: Sagittal x-rays preoperative showing the segmental kyphosis at T11-T12 of 28°. **B**: Sagittal x-rays 3 months postoperative showing a reduction in segmental kyphosis of 10° after implantation of a 55 mm expandable lateral interbody cage (5–20° lordotic) with ALL release and posterior monosegmental fusion. Furthermore, a case vignette of a 59-year-old female with hyperextension fracture AO Spine Type B3 at T11-T12 in ankylosing spondylitis and prior instrumentation from T9-L2 is presented. **C**: In the immediate postoperative images we noticed a large residual gap with 20° lordosis, hindering fusion of the anterior column. **D**: After LLIF T11-T12 with a 55 mm expandable implant (5–20° lordotic) the gap was closed, helping to promote solid fusion.

The trauma patients we included can be divided into two groups, one being those with kyphosing compression or burst fractures and the other being ankylosed patients with AO Spine type B3 extension fractures and large opening gaps. While in the first group, the hyperlordotic cages may be used to help compensate for the loss of SL resulting from the fracture, the cages in the latter group serve to fill out the gap, allowing the adjacent spinal segments to fuse over the large defect caused by the extension trauma. Examples for both patient types are provided in Fig. 3A–D.

LLIF can be an elegant way to address pseudarthrosis while also using this opportunity for segmental correction in patients with previous attempted posterior fusion and varying degrees of hypolordosis (Orita et al., 2018). The virgin access route, large access to the disc space for previous cage extraction, as well as the large footprint LLIF spacer and graft chamber with increased surface area for the interbody fusion facilitate straight-forward procedures (Haider et al., 2022). Despite thorough review of the patient charts and radiological follow-up data, we did not identify any patient with failure of the spacer or pseudarthrosis at the LLIF level during 12 months of follow-up.

In some instances, we also used the expandable LLIF spacers as an intraoperative “backup solution” when insertion of a static LLIF cage had resulted in a violation of the vertebral endplate. After removal of the static cage, the expandable LLIF spacer could then be introduced gently and opened in a controlled manner until a press fit was reached between the non-violated endplate and the pedicle screws of the fractured vertebra.

### 4.2 Benefits and downsides of an expandable LLIF spacer

While many patients can be treated adequately with a static LLIF cage, there may be certain advantages of expandable solutions. The need for necessary trialing and repeated forceful impaction when using static cages is mitigated, as expandable spacers are inserted in a collapsed configuration and expanded in situ until they achieve the desired height and lordosis. Theoretically, this should reduce the risk of endplate damage and cage subsidence – even in patients with a collapsed disc space and/or poor bone quality, which could lead to improved fusion rates (Vaishnav et al., 2020). Depending on the manufacturer, cage height in expandable spacers can be several millimeters higher than in static ones, which can be helpful in patients with high disc space anatomy or otherwise large anterior column defects to close (Macki et al., 2021). Overexpansion and hence -distraction of the intervertebral space may occur with expandable spacers as well, which may lead to endplate injury, unintended ALL rupture and secondary complications such as endplate violation, cage migration, pseudarthrosis and subsidence. Subsidence, in return, may result in loss of lordosis, persistent back or leg pain with poor functional outcome, potentially leading to revision surgery. The most important downside of expandable LLIF spacers is the usually higher implant cost, compared to static cages. As this study did neither include a control group (with static LLIF spacers) nor a cost-effectiveness analysis, we cannot calculate the net benefit of an expandable versus static LLIF spacer from our data.

The actual literature on static or expandable LLIF spacers is limited so far. In 2018, Frisch et al. from the United States (US) published their prospective study of 56 patients undergoing LLIF with either static (n = 29; PEEK TransContinental, Globus Medical, Inc., Audubon, PA) or expandable PEEK cages (n = 27; PEEK CALIBER -L, Globus Medical, Inc., Audubon, PA). The authors found a higher rate of cage subsidence in the static cage group (16.1% vs. 0%, p < 0.01), with all other radiological and clinical outcomes being similar (Frisch et al., 2018). In 2020, Li et al., again from the US, reported a retrospective comparative analysis of 62 consecutive patients with degenerative disc disease undergoing LLIF surgery with static (n = 27; Ti TransContinental, Globus Medical, Inc., Audubon, PA) or expandable (n = 35; Ti RISE-L, Globus Medical, Inc., Audubon, PA) spacers (Li et al., 2020). Patients in the expandable cage group had worse baseline scores on the Oswestry Disability Index (ODI). At 12 months postoperative, both groups showed similar clinical outcomes on the ODI and VAS back/leg metrics without clinically relevant or statistically significant differences (Li et al., 2020). The cage subsidence rates were higher in the static cage group (16.1% vs. 6.7%), but none was symptomatic or needed revision surgery, similar to our series. In 2023, Huo et al. from Australia prospectively compared 50 patients with symptomatic degenerative spondylolisthesis receiving 85 static (Ti Modulus XLIF, NuVasive, Inc., San Diego, CA) and 48 patients receiving 84 expandable cages (Ti RISE-L, Globus Medical, Inc., Audubon, PA) (Huo et al., 2023). They found a higher rate of interbody fusion (94% vs. 82.9%, p = 0.039) in the expandable cage group at 12 months and less cage subsidence at all time points during follow-up, which translated in slightly better VAS scores for back pain and leg pain but similar results on the ODI and SF-12 physical component score at 12 months (Huo et al., 2023). Hiyama et al. from Japan studied the 2-week outcomes of 67 patients undergoing LLIF for lumbar degenerative disc disease with either a static (n = 44; Ti Modulus XLIF or X-TAL cage, NuVasive, Inc., San Diego, CA) or an expandable (n = 23; Ti RISE-L, Globus Medical, Inc., Audubon, PA) spacer (Hiyama et al., 2023). The authors reported no severe AEs including additional surgeries, while radiological (postoperative disc height, SL, foraminal area and dural sac enlargement) and quality outcomes (e.g., operation time and EBL) were similar. Again in 2023, Omosor et al. presented five cases of lumbar degenerative scoliosis and reported the feasibility to treat this condition with LLIF and an expandable spacer (Dual-X LLIF, Amplify Surgical, Inc., Irvine, CA) (Omosor et al., 2023). The small case series did not report long-term clinical or radiological outcomes, but the authors mentioned no cage subsidence in any patient during short-term follow-up. Our own results compare well with these studies, but we did not include a control group with static LLIF cages, as this was not the scope of this work.

### 4.3 Strengths and weaknesses

Our study reports data from a reasonably large, consecutive series of patients treated with innovative technology, which gains increasing popularity, but on which there is limited data (Hiyama et al., 2023; Huo et al., 2023; Macki et al., 2021; Omosor et al., 2023). Despite its retrospective character, the missing data burden was acceptable and we were able to generate solid radiological outcome data. The heterogeneity of the sample in terms of diagnoses for and type of surgery may be considered a weakness, but it best represents the use of these types of implants in the real-world scenario of a tertiary, academic spine center. In contrast, previous studies were confined to very selected populations of patients with degenerative spondylolisthesis,(Huo et al., 2023) degenerative disc disease, (Li et al., 2020) or lumbar degenerative scoliosis (Omosor et al., 2023).

Selection bias is likely to be present in this retrospective study, as we did not control the reasons why patients were receiving an expandable LLIF spacer versus a static LLIF cage or any other type of anterior or posterior interbody fusion. Moreover, the standardized use of patient-reported outcome measures (PROMs) in our center was only introduced in 2022, which is why for this study we classified the functional outcome using the Macnab criteria with a simple 4-tier scale (Macnab, 1971; Maldaner and Stienen, 2020; Tafazal and Sell, 2006). A further limitation of this study is the absence of CT scan images for assessment of the fusion status in all patients, as those are not routinely performed as part of the follow-up regimen in patients faring well. However, in case of unusual pain during follow-up, CT or SPECT scans are ordered to rule out pseudarthrosis. Finally, there was no control group, as it was not the aim of the article to conduct comparative or cost-effectiveness analyses.

### 4.4 Implications for practice

Based on our positive experience with the use of expandable LLIF spacer technology in selected patients over the past years, we will continue to consider it for the mentioned clinical conditions. Considering the favorable radiological and clinical results in this analysis and the available literature, possible extension of its use for other applications is mainly limited by the higher implant price, when compared to the one of static LLIF cages. As more and more spine centers employ the LLIF technique regularly for some applications and gain solid experience in its use, prospective, comparative multi-center studies focusing on well-defined pathologies should be conducted by surgeons with solid experience to generate better evidence regarding the benefit of these innovative compared to conventional techniques. Until then, we recommend considering LLIF with expandable cages as viable alternative to conventional anterior, lateral or posterior interbody fusion devices.

## 5 Conclusions

The use of LLIF with an expandable titanium interbody implant in this series was safe and enabled powerful correction of sagittal segmental cobb angle, which was maintained during follow-up. The rate of patients faring well without evidence of non-union was high (poor outcome in 9.5% at the 3- and 6.3% at the 12-month follow-up). LLIF may be considered for a wide range of conditions, including deformity, trauma, and revision surgery.

## Conflicts of interest

A research grant was provided to the research account of the Kantonsspital St. Gallen, Switzerland, by Globus Medical. The funding source was not involved in the collection of data, analysis or generation of the manuscript but was able to review the paper prior to submission.

## Previous presentations

None.

## Declaration of competing interest

The authors declare the following financial interests/personal relationships which may be considered as potential competing interests: Martin N. Stienen reports financial support was provided by Globus Medical. If there are other authors, they declare that they have no known competing financial interests or personal relationships that could have appeared to influence the work reported in this paper.

## References

[bib1] Amaral R., Marchi L., Oliveira L., Coutinho T., Pimenta L. (2013). Acute lumbar burst fracture treated by minimally invasive lateral corpectomy. Case Rep. Orthop..

[bib2] Charlson M.E., Pompei P., Ales K.L., MacKenzie C.R. (1987). A new method of classifying prognostic comorbidity in longitudinal studies: development and validation. J. Chron. Dis..

[bib3] Frisch R.F., Luna I.Y., Brooks D.M., Joshua G., O'Brien J.R. (2018). Clinical and radiographic analysis of expandable versus static lateral lumbar interbody fusion devices with two-year follow-up. J. Spine Surg..

[bib4] Haider G., Wagner K.E., Chandra V., Cheng I., Stienen M.N., Veeravagu A. (2022). Utilization of lateral anterior lumbar interbody fusion for revision of failed prior TLIF: illustrative case. J. Neurosurg Case. Lessons.

[bib5] Hiyama A., Katoh H., Sakai D., Sato M., Watanabe M. (2023). Early radiological assessment of static and expandable cages in lateral Single position for indirect decompression- lateral lumbar interbody fusion. World Neurosurg..

[bib6] Huo C.W., Malham G.M., Biddau D.T., Chung T., Wang Y.Y. (2023). Lateral lumbar interbody fusion using expandable vs static titanium interbody cages: a prospective cohort study of clinical and radiographic outcomes. Internet J. Spine Surg..

[bib7] Joswig H., Hock C., Hildebrandt G., Schaller K., Stienen M.N. (2016). Microscopic lumbar spinal stenosis decompression: is surgical education safe?. Acta Neurochir..

[bib8] Laouissat F., Sebaaly A., Gehrchen M., Roussouly P. (2018). Classification of normal sagittal spine alignment: refounding the Roussouly classification. Eur. Spine J..

[bib9] Leveque J.C., Yanamadala V., Buchlak Q.D., Sethi R.K. (2017). Correction of severe spinopelvic mismatch: decreased blood loss with lateral hyperlordotic interbody grafts as compared with pedicle subtraction osteotomy. Neurosurg. Focus.

[bib10] Li Y.M., Frisch R.F., Huang Z., Towner J., Li Y.I., Greeley S.L., Ledonio C. (2020). Comparative effectiveness of expandable versus static interbody spacers via MIS LLIF: a 2-year radiographic and clinical outcomes study. Global Spine J..

[bib11] Macki M., Hamilton T., Haddad Y.W., Chang V. (2021). Expandable cage technology-transforaminal, anterior, and lateral lumbar interbody fusion. Oper Neurosurg. (Hagerstown).

[bib12] Macnab I. (1971). Negative disc exploration. An analysis of the causes of nerve-root involvement in sixty-eight patients. J. Bone Joint Surg. Am..

[bib13] Maldaner N., Stienen M.N. (2020). Subjective and objective measures of symptoms, function, and outcome in patients with degenerative spine disease. Arthritis Care Res..

[bib14] Meredith D.S., Kepler C.K., Huang R.C., Hegde V.V. (2013). Extreme lateral interbody fusion (XLIF) in the thoracic and thoracolumbar spine: technical report and early outcomes. HSS J..

[bib15] Mundis G.M., Turner J.D., Kabirian N., Pawelek J., Eastlack R.K., Uribe J., Klineberg E., Bess S., Ames C., Deviren V., Nguyen S., Lafage V., Akbarnia B.A., International Spine Study G. (2017). Anterior column realignment has similar results to pedicle subtraction osteotomy in treating adults with sagittal plane deformity. World Neurosurg..

[bib16] Omosor E., Edelbach B.M., Amer H., Hussain N.S. (2023). Utilization of dual expandable cages in lateral lumbar interbody fusion surgery. Cureus.

[bib17] Orita S., Nakajima T., Konno K., Inage K., Sainoh T., Fujimoto K., Sato J., Shiga Y., Kanamoto H., Abe K., Inoue M., Kinoshita H., Norimoto M., Umimura T., Aoki Y., Nakamura J., Matsuura Y., Kubota G., Eguchi Y., Hynes R.A., Akazawa T., Suzuki M., Takahashi K., Ohtori S. (2018). Salvage strategy for failed spinal fusion surgery using lumbar lateral interbody fusion technique: a technical note. Spine Surg. Relat. Res..

[bib18] Pizones J., Moreno-Manzanaro L., Sanchez Perez-Grueso F.J., Vila-Casademunt A., Yilgor C., Obeid I., Alanay A., Kleinstuck F., Acaroglu E.R., Pellise F., Group E.E.S.S. (2020). Restoring the ideal Roussouly sagittal profile in adult scoliosis surgery decreases the risk of mechanical complications. Eur. Spine J..

[bib19] Podet A.G., Morrow K.D., Robichaux J.M., Shields J.A., DiGiorgio A.M., Tender G.C. (2020). Minimally invasive lateral corpectomy for thoracolumbar traumatic burst fractures. Neurosurg. Focus.

[bib20] Raizman N.M., O'Brien J.R., Poehling-Monaghan K.L., Yu W.D. (2009). Pseudarthrosis of the spine. J. Am. Acad. Orthop. Surg..

[bib21] Roblesgil-Medrano A., Tellez-Garcia E., Bueno-Gutierrez L.C., Villarreal-Espinosa J.B., Galindo-Garza C.A., Rodriguez-Barreda J.R., Flores-Villalba E., Eugenio Hinojosa-Gonzalez D., Figueroa-Sanchez J.A. (2022). Thoracolumbar burst fractures: a systematic review and meta-analysis on the anterior and posterior approaches. Spine Surg. Relat. Res..

[bib22] Rockwood K., Song X., MacKnight C., Bergman H., Hogan D.B., McDowell I., Mitnitski A. (2005). A global clinical measure of fitness and frailty in elderly people. CMAJ (Can. Med. Assoc. J.).

[bib23] Saigal R., Mundis G.M., Eastlack R., Uribe J.S., Phillips F.M., Akbarnia B.A. (2016). Anterior column realignment (ACR) in adult sagittal deformity correction: technique and review of the literature. Spine.

[bib24] Sebaaly A., Grobost P., Mallam L., Roussouly P. (2018). Description of the sagittal alignment of the degenerative human spine. Eur. Spine J..

[bib25] Smith W.D., Dakwar E., Le T.V., Christian G., Serrano S., Uribe J.S. (2010). Minimally invasive surgery for traumatic spinal pathologies: a mini-open, lateral approach in the thoracic and lumbar spine. Spine.

[bib26] Sterne J.A., White I.R., Carlin J.B., Spratt M., Royston P., Kenward M.G., Wood A.M., Carpenter J.R. (2009). Multiple imputation for missing data in epidemiological and clinical research: potential and pitfalls. BMJ.

[bib27] Tafazal S.I., Sell P.J. (2006). Outcome scores in spinal surgery quantified: excellent, good, fair and poor in terms of patient-completed tools. Eur. Spine J..

[bib28] Terrapon A.P.R., Zattra C.M., Voglis S., Velz J., Vasella F., Akeret K., Held U., Schiavolin S., Bozinov O., Ferroli P., Broggi M., Sarnthein J., Regli L., Neidert M.C. (2021). Adverse events in neurosurgery: the novel therapy-disability-neurology grade. Neurosurgery.

[bib29] Vaccaro A.R., Oner C., Kepler C.K., Dvorak M., Schnake K., Bellabarba C., Reinhold M., Aarabi B., Kandziora F., Chapman J., Shanmuganathan R., Fehlings M., Vialle L., Injury A.O.S.C., Trauma Knowledge F. (2013). AOSpine thoracolumbar spine injury classification system: fracture description, neurological status, and key modifiers. Spine.

[bib30] Vaishnav A.S., Saville P., McAnany S., Kirnaz S., Wipplinger C., Navarro-Ramirez R., Hartl R., Yang J., Gang C.H., Qureshi S.A. (2020). Retrospective review of immediate restoration of lordosis in single-level minimally invasive transforaminal lumbar interbody fusion: a comparison of static and expandable interbody cages. Oper Neurosurg. (Hagerstown).

[bib31] Walker C.T., Xu D.S., Godzik J., Turner J.D., Uribe J.S., Smith W.D. (2018). Minimally invasive surgery for thoracolumbar spinal trauma. Ann. Transl. Med..

[bib32] Xu D.S., Paluzzi J., Kanter A.S., Uribe J.S. (2018). Anterior column release/realignment. Neurosurg. Clin..

